# Foreign Quacks and American Medical Colleges

**Published:** 1876-04

**Authors:** 


### Editorial.

### Foreign Quacks and American Medical Colleges.

By reason of the ready sale which the diplomas of one or two so-called American Medical Colleges realized in Europe, the impression seems to prevail there that the only thing necessary to obtain an American Degree of M. D. is to forward the requisite amount of cash.

The Dean of the Medical Department of the University of Buffalo received, some time since, a letter from a lady practitioner in England, from which we make the following extract:

*To the Dean of the Faculty, University of Buffalo:*

SIR: I shall feel obliged by your kindly informing me if I can obtain a diploma from your College by passing an examination in England. It appears feasible to me that one or more gentlemen in the profession might be appointed to report upon my education and practical experience in midwifery. It is impossible for me to leave England for a *viva voce* examination, as I should lose the appointments I now hold. I may say that Dr. K——, of Bulstrode street, in London (an examiner at the Apothecaries' Hall, one of the most honored medical institutions in England), and several others, would undertake to examine me, if you do not consider my professional attainments sufficient. The copies of my certificates and testimonials I now enclose. Any of the gentlemen to whom I refer will reply to any enquiries you may deem it expedient to make; and I shall be happy to remit the fees to any one you may appoint in America.

I should not take the step of thus appealing to your College for assistance, but that the Colleges in England have not as yet afforded us facilities for a free entrance to their examinations, as you will see by the enclosed letter from the Royal College of Surgeons. * * * * * *
In conclusion, may I beg the favor of your informing me if the Faculty of your College confers the degree of Doctor of Medicine, and what would be the amount of fees upon taking up such degree?

I am, Sir, Yours Obediently,

——— ————.

We do not copy this letter because it is anything rare ; on the contrary, we are sorry to say that such is the reputation, which the disgraceful traffic in medical degrees, by one or two bogus colleges, has entailed upon American Colleges, that the opinion has gained ground abroad that a degree can be obtained on like terms from all. There are few Colleges, we presume, which have not received propositions similar to this.

The lady from whom this letter was received presents some very good credentials, endorsed by men known in America as well as in England. She seems to have attained a very flattering recognition of her talents as a practitioner of midwifery, both in private practice and as attendant to hospitals and dispensaries. Hitherto, however, she has practiced without a diploma, and being unable to obtain one in England, she turns in her anxiety to America to furnish the much-desired parchment. To the above letter Dr. Rochester replied as follows :

MEDICAL DEPARTMENT,
UNIVERSITY OF BUFFALO.
BUFFALO, N. Y., April 5, 1876.

*Miss* ——— —————,

MADAM : Your two communications were received a month apart. I have the honor of informing you that the Medical Department of the University of Buffalo confines its degrees to its own matriculants, who have studied medicine for three or more years under its direction, and within its walls, and under no circumstances does it *sell a diploma.*

Your Obt. Servt.,

THOS. F. ROCHESTER,

Dean of the Faculty.

The fact that any medical college should find it necessary to deny that it sells a diploma, is, of itself, a humiliating one to all who have the interest of medical education at heart. We hear yearly more or less of a cry for the advancement of the standard of medical education ; but the cry is generally made by those who know nothing of the difficulties of the task they impose. Prompted more by a feeling of envy and a desire to accomplish the overthrow and discomfiture of those who, by reason of holding professional chairs, have inspired their envy and malice, there are those who cry out against the present low grade, but do nothing to help build it up.

The day is not far distant, we hope, when students will be compelled to *study* medicine for *for three or more* years, *and within the walls and under the direction of* some medical college ; and not as is now the case, attend a short course of lectures in the winter and teach school or lounge about some doctor's office in the summer. When the medical colleges can take the matter of medical education entirely out of the hands of physicians, and ensure *three* or *more years* of well-directed study, properly graded, the standard of medical education will be elevated, and not until then. Then the student will have the advantages of recitations, laboratory work, and complete clinical instruction in hospitals. Now his advantages consist of the use of a few text-books in the office of his preceptor, laboratory work in the way of compounding fever mixtures and making pills, and clinical opportunities in the shape of an occasional case of typhoid fever or a broken arm. Under these circumstances, how can the medical graduates of our colleges be other than what they are? If the material sent by the physicians is crude, the result can be naught but imperfect. If the profession will as a unit support the faculties of our colleges in adopting a graded course, and will discourage any young man from commencing the study of medicine who is not qualified so to do, and who cannot spend the time and money to thoroughly qualify himself for a degree, the elevation of the standard will be an easy task; but if envy and malice are to continually throw obstructions in the path, the task will be of a different nature.

Foreigners are beginning to look with a large degree of interest upon the establishment of graded courses in some of our medical schools. They are looking upon it with interest for the reason that American diplomas have hitherto been easy of attainment by the quacks with which Europe, as well as America, is infested.

